# 

**Published:** 1869-01

**Authors:** 


### Books and Pamphlets Received.

On the Dynamics, Principles and Phylosophy of Organic Life; an effort to obtain definite conceptions of how do medicines produce their effects? By Z. C. McElroy, Zanesville, Ohio.

Svapnia, or Purified Opium, consisting of Meconates of Morphia, Codeia and Narceia, and made by assay. An important substitute for Morphia and Crude Opium. By Dr. J. M. Bigelow, Detroit.

Scirrhus, or Malignant Disease of the Rectum. By Alden March, M. D., Albany.

An Intra-Mural Fibrous Tumor removed from the Anterior Wall of the Uterus. By William H. Byford, A. M., M. D., Professor of Obstetrics, etc., in Chicago Medical College.

Report on Insanity. By Charles Alfred Lee, M. D., Peekskill, N. Y.

Thirteenth Annual Report of the Trustees of the State Lunatic Hospital at Northampton, Mass.

